# Iatrogenic Side Effects of Pain Therapies

**DOI:** 10.7759/cureus.44583

**Published:** 2023-09-02

**Authors:** Christopher Gharibo, Asbjørn M Drewes, Frank Breve, Martina Rekatsina, Marco Antonio Narvaez Tamayo, Giustino Varrassi, Antonella Paladini

**Affiliations:** 1 Pain Management, NYU Langone Health, New York City, USA; 2 Gastroenterology and Hepatology, Aalborg University Hospital, Aalborg, DNK; 3 Pharmacy, Temple University, Philadelphia, USA; 4 Anesthesia and Pain Management, University of Athens, Athens, GRC; 5 Pain Medicine, Hospital Obrero, La Paz, BOL; 6 Pain Medicine, Paolo Procacci Foundation, Rome, ITA; 7 Life, Health and Environmental Sciences (MESVA), University of L'Aquila, L'Aquila, ITA

**Keywords:** opioid induced constipation, opioids side effects, interventional pain management, side effects of analgesics, polypharmacy, pain control, opioids, adverse effects, analgesia

## Abstract

Pain regimens, particularly for chronic cancer and noncancer pain, must balance the important analgesic benefits against potential risks. Many effective and frequently used pain control regimens are associated with iatrogenic adverse events. Interventional procedures can be associated with nerve injuries, vascular injuries, trauma to the spinal cord, and epidural abscesses. Although rare, these adverse events are potentially catastrophic. Pharmacologic remedies for pain must also consider potential side effects that can occur even at therapeutic doses of over-the-counter remedies such as paracetamol (acetaminophen) or nonsteroidal anti-inflammatory drugs. Opioids are effective pain relievers but are associated with many side effects, some of which can be treatment limiting. A prevalent and distressing side effect of opioid therapy is constipation. Opioid-induced constipation is caused by binding to opioid receptors in the gastrointestinal system, making conventional laxatives ineffective. Peripherally acting mu-opioid receptor antagonists are a new drug class that offers the benefits of preserving opioid analgesia without side effects in the gastrointestinal system. An important safety concern, particularly among geriatric patients is the increasingly prevalent condition of polypharmacy. Many senior patients take five or more medications, including some that may be contraindicated in geriatric patients, duplicative of other drugs, have potential pharmacokinetic drug-drug interactions, or may not be the optimal choice for the patient’s age and condition. Careful assessment of medications in the elderly, including possibly deprescribing with tapering of certain drugs, may be warranted but should be done systematically and under clinical supervision.

## Introduction and background

Pharmacologic therapy and interventional procedures intended to mitigate or eliminate pain may cause treatment-limiting or even potentially life-threatening side effects [1]. Polypharmacy has been associated with risks of drug-drug interactions, opioid-associated side effects can be distressing, and interventional procedures have been linked to rare but potentially severe adverse effects [2, 3]. For chronic cancer or noncancer pain patients, pain care has become a trade-off: Accept the analgesic benefits and suffer the side effects or forego pain relief in order to avoid the side effects? All pain treatments confer some degree of risk, which must be interpreted individually for each patient, and both pharmacologic and interventional pain treatments must be carefully weighed in terms of relative risk, desired benefits, and potential adverse events. Pain is associated not just with disease and injury but also with medical care such as surgery or analgesics. Ideally, analgesia should not be associated with iatrogenic adverse effects.

The management of iatrogenic effects of pain therapy is an important consideration for pain medicine that must be subjected to both clinical judgment and patient preferences. Clinicians focusing on the medical goal of reducing intractable pain may trivialize side effects that the patient finds diminish his quality of life. Oncologists battling cancer may minimize the suffering of painful chemotherapy-induced neuropathy. Nothing is more personalized in medicine than an individual’s determination of what constitute acceptable risks and tolerable therapy. Clinicians may sometimes be surprised by a patient who opts to discontinue opioids and “live with the pain,” just as they may not understand a patient who seeks strong pain control for what could be considered manageable pain.

Physicians must be aware of the risks and benefits of the drugs and interventions they recommend and take steps to educate their patients about such risks and benefits. No analgesics or pain interventions are without risk, and clinicians must become partners rather than patriarchs as they help their patients weighing out risks versus benefits.

This article is based on a series of presentations from the “Past, Present and Future in Pain Medicine” held in Tunis, Tunisia on May 11-13, 2023 about iatrogenic side effects of pain-control interventions and drug therapy. It is a short review of various salient topics on the subject of iatrogenic issues in pain management and are based on the current literature and the clinical experiences and academic expertise of the authors.

## Review

Iatrogenic injuries in interventional pain care

Iatrogenic injuries related to interventional pain procedures are often multifactorial, tracing back to factors related to procedural techniques, communications among the medical team, and poor clinical judgement [4]. Needle-related injuries in epidural injections are rare but can be catastrophic. Direct needle trauma to a nerve or the spinal cord during cervical injections to treat chronic pain is the most common procedure-related event for cervical chronic pain malpractice in the United States [4, 5]. Needle-related injuries can also be due to “jetting” or the sudden high-velocity injection of a drug, runoff that can lead to adhesions, and pressurization caused by the injectate that blocks or interrupts the flow of cerebrospinal fluid. Dural puncture occurs in about 1.4% of cases and most frequently occurs at C5/6 and C7-C11 [6].

While the shape of the needle is often discussed in terms of avoiding injury, there is a paucity of evidence supporting the alleged safety advantages of blunt needles [7]. Clinical experience has shown that blunt needles do not offer good directionality and may prolong procedures. A case report in the literature has found that a blunt needle, negative aspiration, or a test dose of the local anesthetic does not offer reliable protection against vascular penetration [8]. However, in a retrospective study of 185 transforaminal epidural steroid injections, paresthesia was reported in three cases with blunt needles versus eight cases with a sharp needle. In this same study, vascular penetration occurred in two and 13 cases of blunt and sharp needles, respectively [9]. However, in a study of intravascular injection rates during transforaminal epidural steroid injections using blunt and sharp needles (n=108), blunt needles did not reduce intravascular injection but prolonged the procedure time [10]. Thus, the evidence at this time is equivocal and limited regarding the superiority of blunt versus sharp needles.

Steroids themselves may be associated with adverse events, to the point that the use of epidural steroids in and of itself is coming under question because of the rare but potential risk for stroke, vision loss, paralysis, and fatality [11]. The literature has also reported cases of compounded corticosteroids contaminated with fungal meningitis [4, 12]. Infarction with epidural injections might occur when particulate steroids are injected, a vessel is dissected, trauma to an artery occurs, or an arterial muscle spasm happens [4]. Dexamethasone is a non-particulate corticosteroid but has also been associated in case reports with spinal infarction [4, 13]. Particulate steroid aggregation, mechanical disruption of the vessels, and/or thrombotic occlusion can cause an adverse event, either near the injection site or distal from it. Occlusion and mechanical disruption are the main sources of vessel-related nerve injuries [4].

The anatomy of the epidural space is complex, with considerable interpatient variability. Many patients have a ligamentum flavum with midline gaps of about 1 mm which can allow for a small amount of leakage of injectate [14]. Such gaps occur mainly between C3/4 to T2/3; between T10/11 and L1/2; or at lower lumbar ranges [15]. Epidural injectates are redistributed from the epidural space via the meninges into cerebrospinal fluid and from there, to their targets [16]. Interruptions in the ligamentum flavum are believed to occur in more than half of patients and usually appear in the cervical region, above C7-T1 but may continue through T3/4 [15]. For patients with ligamentum flavum gaps, the standard of care for neuraxial injections demands fluoroscopic guidance, particularly lateral views [17]. Interventional pain specialists gain access to the epidural space using a caudal, transforaminal, or interlaminar technique. When injecting corticosteroids, the transforaminal approach is associated with a higher rate of post-injection adverse events because of potential damage to the local vasculature [4]. In addition to technique-related complications, steroids themselves are associated with adverse events, both due to particulates and pharmacological effects, such as hypothalamic-pituitary-adrenal suppression and immune system suppression [4].

An epidural abscess with or without meningitis may also occur after corticosteroid injections, such as those for radicular back pain [18]. Paraspinal, peridural, or spinal injections corticosteroid injections combined with analgesics can confer a small but potentially life-threatening risk of para-meningeal inoculation of bacteria. Spinal epidural abscesses occur approximately 12 times per 100,000 hospital admissions [19]. While a rare iatrogenic complication, such events may cause a large subset of community-acquired purulent central nervous system infections [20]. In an observational study of 128 patients, the mean age at onset of epidural abscess was 54 years, the mean number of injections was 2.5, and the time lapse between the injection and onset of symptoms was between 2 days and 4 months; excluding the outlier value, the range would be 2 to 7 days [20]. Prophylactic antibiotic treatment for *Staphylococcus aureus* may be helpful when immunocompromised patients must undergo epidural corticosteroid injections [18].

Conus medullaris infarction is a rare but potentially catastrophic complication related to interlaminar epidural steroid injections [21]. The conus medullaris is a cone-shaped structure made up of the sacral and coccygeal portions of the spinal cord, usually starting at around L1 in adults. Nerves that serve the lower extremities, bowels, and bladder originate in the conus medullaris. Vascular injury leading to conus medullaris infarction may be triggered by vasospasm, thrombus, dissection, or embolization of the vessel caused by particulate steroids [13]. A case report describes the abrupt onset of numbness and weakness in the lower extremities followed by incontinence after an L4 transforaminal epidural dexamethasone injection [13]. The patient was 60 years old and this was the first such report known to the authors of conus infarction in a patient administered dexamethasone. The patient had previously had a similar injection with no adverse events [13].

Vascular nerve injury caused by injections may be due in part to the anatomical variations of vasculature among patients. Furthermore, tortuous or unusual vessels can make injections challenging. Vessel occlusion can directly injure a nerve. Nerve injury may also be caused by certain steroids, a newly formed thrombus, atherosclerotic plaque, dissection, retrograde flow, and other conditions. The mechanical disruption of the vasculature, such as might be caused by needle transection, can also cause nerve damage. In a 17-year retrospective study of 354 iatrogenic nerve injury cases associated with an injection, the presenting symptoms were pain, paresthesia, and sensory-motor dysfunction. This study reported that 46.3% of nerve-injured patients did not recover [22].

Pharmacologic therapy

Analgesics and Iatrogenic Adverse Events

Drugs remain the mainstay in the armamentarium against pain [23]. Nonsteroidal anti-inflammatory drugs (NSAIDs) can be effective pain relievers but may result in gastrointestinal side effects. Selective cyclooxygenase (COX) inhibitors may, on the other hand, have cardiovascular side effects [24]. In some patients, NSAIDs may promote ulcers or enteropathy in the small intestine and colon, and paracetamol (acetaminophen) can be hepatotoxic at supratherapeutic doses [25, 26]. Gabapentinoids have been associated with changes in motility in the intestine, acetyl salicylic acid may cause ulcers or enteropathy [27]. Opioids are effective pain relievers, but they can adversely affect the motility of the esophagus, stomach, and intestinal system and cause constriction of the anal sphincter [28]. Opioids also interfere with fluid processing in the gut resulting in dry feces that is difficult to evacuate [29]. In addition, opioids can adversely alter liver metabolic processes [30]. Other side effects associated with opioids include somnolence, nausea, vomiting, dizziness, pruritus, respiratory depression, and the risks of opioid use disorder, tolerance, and dependence [2, 31]. These side effects can be treatment limiting and have limited the prescribing of opioids to inappropriate patients [23]. The determination of the intensity, severity, and tolerability of opioid-associated adverse events is based on the patient’s subjective impressions. While validated risk tools exist to help measure pain intensity, there is no similar instrument that can help capture the effects of opioid-associated side effects on patients.

Opioid-induced constipation (OIC) is considered one of the most prevalent and most distressing of all opioid-associated side effects [32]. Resulting from the activity of opioids mainly on the mu-opioid receptors in the gut, OIC differs fundamentally from other forms of constipation and can be refractory to conventional constipation treatments [33]. The Rome IV diagnostic criteria provide an assessment tool for evaluating OIC and its effects on patients in real-world clinical practice [34]. While these new Rome IV diagnostic criteria are useful in assessing patients with more severe forms of OIC, they may not be as serviceable for patients with milder symptoms [34]. The Bowel Function Index (BFI) may be better used for evaluations, as it offers well-defined cutoff values and has been translated into many languages [35]. Over-the-counter and prescription laxatives intended to treat occasional constipation are a mechanistic mismatch for OIC [36]. OIC is known to adversely impact lifestyle, wellbeing, activities of daily living, quality of life, and even work productivity [36]. Unlike certain other opioid-associated side effects such as dizziness or nausea, OIC does not diminish over time and with increasing opioid tolerance.

Opioids can delay gastric emptying and peristalsis in the gastrointestinal system, resulting in slower absorption of medication and increased absorption of fluid. This increased fluid absorption denies water to the gastrointestinal system, causing hard, dry stools that are difficult to pass. Opioids also increased the tone of the anal sphincter and interfere with the body’s normal reflexes regarding defecation [37-39]. Opioids are known to dysregulate and interfere with anal sphincter function, which can cause symptoms of straining or incomplete evacuation. A clinical study of an opioid-induced bowel disorder in healthy subjects (n=24) found that naloxegol relaxed the anal sphincter by 15% and improved symptoms [38].

The incidence of OIC among patients taking opioid therapy ranges from 15% to 81%. In a survey in Japan of patients taking analgesics, constipation was reported by 34% of those taking opioids and 29% of those taking nonopioid pain relievers. However, more respondents in the opioid group fulfilled two or more Rome IV diagnostic criteria for constipation (28% vs. 19%) [40]. This wide range is due to the lack of an expert consensus definition of OIC and how it is to be measured [41]. It has been reported that opioid-associated side effects are the main reason for opioid discontinuation for 19% of patients prescribed opioids [42]. A survey of 322 patients taking opioids and laxatives every day found that 81% of respondents reported constipation and that, of all opioid-associated side effects, constipation was considered the most bothersome [43]. In a survey of pain patients on opioid therapy (25% cancer pain, 75% noncancer pain), 66% reported OIC, of whom 41% stated they were satisfied with how their OIC was being clinically managed. However, many opioid patients still struggle with OIC. In an effort to manage OIC on their own, 50% of noncancer pain patients taking opioids said they reduced their opioid dose [44].

In a study of 25 healthy subjects taking oral oxycodone or placebo over five days in a double-blind crossover study, it was found that oxycodone significantly prolonged the median total gastrointestinal transit time from 22.2 hours to 43.9 hours [45]. Opioids decrease intestinal secretions [46]. In a survey of 557 respondents with chronic constipation, the most prevalent symptom reported was straining to defecate and the symptoms considered most severe were straining, bloating, and hard stools [47]. Over half of respondents (52%) stated that constipation adversely affected their quality of life, led to decreased productivity in 12%, and was responsible for a mean of 2.4 days of absence in the past 30 days. In this survey, most respondents (96%) had used or were currently using some sort of treatment to relieve their constipation, but 47% were not satisfied with the results. This lack of satisfaction with constipation therapy was mainly due to poor efficacy (82%) and concerns about safety (16%) [47]. It is important to note that this survey included people with chronic constipation regardless of its etiology and included, but was not limited to, opioid patients.

The prevalence of constipation symptoms increased with the duration of opioid therapy; health-related quality of life was low in those with chronic abdominal pain [48]. In a survey of opioid patients, of those respondents who met the Rome IV qualifications for OIC (n=951), more than half reported their most frequent symptoms were: straining to defecate, a sensation of blockage, abdominal bloating, hard or lumpy stools, feeling of incomplete evacuation, stomach cramps, rectal burning during or after bowel movements, and hemorrhoids. Similar symptoms, but to a lesser extent, occurred in opioid therapy patients who did not meet Rome IV criteria for OIC. For example, the most common symptom in the latter subpopulation was abdominal bloating (59%), reported by 83% of those who met the Rome IV criteria .

From a large multinational European survey, OIC was a bothersome side effect for those taking weak (38%) and strong (40%) opioids, although those using strong opioids reported more intense symptoms [34]. Symptoms reported by weak and strong opioid users were similar. Most frequently reported by both groups were straining to defecate, abdominal bloating, and the sensation of bowel blockage, but there was less impact on quality of life among those OIC patients taking weak opioids [34].

In a survey of opioid patients, 41% of respondents said that their prescriber had told them about the possibility of OIC, 29% recommended a high-fiber diet and exercise while taking opioids, and only 20% were prescribed a laxative with their opioid analgesics [34]. Once on an opioid regimen, some respondents stated they found pain more tolerable than OIC [49]. However, some opioid patients are reluctant to discuss constipation with their clinical team, perhaps because of personal embarrassment or the belief that little can be done to relieve it [50].

The manner in which OIC is treated appears to vary among specialists. In a survey of 501 various specialists who had occasion to treat patients dealing with OIC, 60% expressed high interest in the topic of OIC, particularly pain specialists and anesthesiologists, while psychiatrists expressed moderate interest. In this survey, oncologists and palliative care specialists were the most likely to treat OIC [32]. Diagnosis mainly relied on patient diaries, and the Rome IV criteria, the bowel function index, or the Bristol stool scale was used by fewer than 10% of physicians [32]. Despite this, 99% of respondents agreed with the statement that OIC could affect a patient’s quality of life [32]. Ten percent of physician respondents thought knowledge of possible OIC might worry patients, and, for that reason, did not mention OIC unless the patient brought up the topic, typically once symptoms occurred [32].

OIC must be recognized as a prevalent and important adverse effect of opioid treatment and physicians must be prepared to offer meaningful treatment options [51]. In diagnosing OIC, it is important to differentiate between true OIC and what might be called opioid-exacerbated constipation. OIC relates to the opioid receptor in the gastrointestinal tract. Opioid-exacerbated constipation may be due to one or more of the following: other drugs the patient is taking, diminished activity level due to illness or injury, poor diet or limited food intake, and underlying disease [51, 52]. Prolonged exposure is not necessary for OIC, which can develop after a brief course of opioids and should be considered even for patients who are administered opioids for a few days to manage acute pain. However, OIC is more prevalent among those with prolonged exposure, and it is best to inform and work with such patients to manage these side effects proactively rather than leave patients to figure out their own ways of dealing with this symptom [51].

While a conventional laxative may be a good first-line approach to OIC, if that is not effective, a peripherally acting mu-opioid receptor antagonist (PAMORA) may be considered [51]. A PAMORA provides the benefits of opioid analgesia without the gut-related side effects. PAMORAs must be considered as an option to allow the patient the benefits of effective analgesia with diminished or eliminated risk of OIC. PAMORA products include methylnaltrexone, naloxegol, and naldemedine, all of which act on the mu-opioid receptors of the gut [53]. Naldemedine was based on naltrexone but structurally altered to prevent it from passing the blood-brain barrier [54]. Naloxegol is a PEGylated form of the drug naloxone and its large molecules are generally unable to cross the blood-brain barrier, meaning that naloxegol does not exert an effect on the opioid in the central nervous system [55]. Naldemedine likewise was based on naltrexone but structurally altered to prevent it from passing the blood-brain barrier [54]. Patients initiating opioid therapy may be started on a PAMORA or patients may be rotated from a conventional laxative to a PAMORA to manage OIC symptoms.

Patients taking opioids may be advised to start at the outset to commence a bowel regimen, make lifestyle changes in terms of exercise and a high-fiber diet, and take a conventional laxative as needed. The problem with this advice is that it may not address OIC; the mechanisms of action of conventional laxatives, such as osmotic effects, motor effects, lubrication benefits, or effects on the microbiota, are not helpful in reducing OIC. On the other hand, conventional laxatives have several advantages in that they are relatively cheap, available over the counter, and are familiar to most patients. For that reason, conventional laxatives are often recommended as a first-line therapy before OIC symptoms are confirmed. A retrospective multicenter study of 619 hospitalized cancer patients receiving opioid analgesics found a significantly lower incidence of constipation in those patients who were prescribed prophylactic laxatives compared to those were did not receive laxatives (34% vs. 55%, respectively, p<0.001) [56]. Over the long term, and if true OIC develops, conventional laxatives will not be helpful. A pilot study of 24 opioid patients taking standard laxative therapy found that 43% did not respond to laxatives and, if the patients who developed diarrhea were counted as non-responders, the nonresponse rate was 75% [57].

Polypharmacy

Polypharmacy refers to the consistent use of multiple concurrent medications, a condition that is particularly prevalent among geriatric patients. Few disease-specific guidelines address the management of comorbidities; there is little guidance in prescribing for multimorbid patients; and there is a paucity of deprescribing protocols to address the reduction of polypharmacy [3]. Even a universal definition of polypharmacy is elusive. The search for a consensus definition of polypharmacy was the subject of a systematic review that examined 110 articles on the topic and found that 80% of articles defined polypharmacy strictly in numerical terms, ranging from ≥ 2 to ≥ 11 drugs, with the most frequent definition ≥ 5 drugs (46%). Only 15 articles mentioned duration as a consideration in the definition and few articles attempted to distinguish between appropriate and inappropriate polypharmacy [58]. In another review, investigators found 143 definitions of polypharmacy and related terms, noting that some definitions varied by patient age and hospitalization status [59].

Using the definition of taking ≥ 5 concurrently prescribed drugs, irrespective of patient age or duration of therapy, polypharmacy is prevalent and increasing. The potential risks of polypharmacy include drug-drug interactions, drug-food interactions, poor adherence, adverse drug reactions, falls, frailty, increased costs, and patient confusion [60]. Many geriatric patients take both opioids, for pain, and benzodiazepines, for insomnia and/or anxiety, but both are central nervous system depressants and may exert potent respiratory depressive effects in combination [61]. A particular concern has been described as “escalating polypharmacy,” which occurs as patients are given new prescriptions for new complaints rather than being subjected to a review of currently prescribed medications [60, 62]. Another concern is the “prescribing cascade” in which an adverse drug reaction from one drug is noted but misdiagnosed as a new condition, resulting in a new medication being prescribed. For example, a hypertensive patient is prescribed a calcium-channel blocker; should this result in the side effect of peripheral edema, the peripheral edema could be misdiagnosed as a new condition for which diuretics are prescribed [63]. Prescribing cascades are not unusual, particularly in light of busy clinical practice and rapid patient throughput. Many geriatric patients on polypharmacy might benefit from deprescribing, but an obvious shortfall here is the lack of expert guidance about deprescribing, particularly for multimorbid geriatric patients [64].

Polypharmacy often occurs in patients who have more than one chronic condition, more than one physician, and/or no primary care physician. This is common in older patients who develop degenerative age-related conditions and often suffer from chronic pain. While opioids are effective pain relievers, current guidance suggests that alternatives be tried as first-line therapy and if opioids are needed that they be used at the lowest effective doses for the shortest duration of time possible [61]. Not all patients with chronic pain are appropriate candidates for opioid analgesics, in which case, multimodal analgesic approaches, nonopioid pharmacologic treatment, and nonpharmacologic approaches may be warranted. Dose reductions may be necessary due to age-related changes in kidney or liver function.

The Beers criteria, the Screening Tool of Older Person’s Prescriptions-Screening Tool to Alert to Right Treatment (STOPP-START) criteria, and the anticholinergic drug scale are important resources in combating potentially dangerous polypharmacy in geriatric patients. The Beers criteria are evidence-based guidance about specific drugs that may cause side effects, be contraindicated, or may have better and/or safe alternatives for geriatric patients. The Beers criteria are endorsed by the American Geriatric Society [65, 66]. Among the numerous analgesic and anti-inflammatory agents to be avoided by older patients mentioned in the Beers criteria are meperidine, etodolac, ibuprofen, ketorolac, and naproxen among others. The Beers criteria instead suggests the use of paracetamol, capsaicin, the lidocaine patch, topical nonsteroidal anti-inflammatory drugs, and other substitutes [66].

The STOPP-START criteria makes recommendations based on expert consensus and screens for drug-drug interactions, duplicative treatments, and potentially inappropriate medications. STOPP-START guidance is considered particularly appropriate for multimorbid seniors [67]. The anticholinergic drug scale lists 217 medications with established anticholinergic effects that may be inappropriate for older patients as these drugs may cause cognitive and/or functional deficits, falls, morbidity, and mortality [68]. This list includes opioids, such as codeine, oxycodone, tramadol, hydrocodone, and others. The known side effects of anticholinergic agents range from the mild, such as dry mouth, constipation, dilated pupils, blurred vision, tachycardia, and decreased sweating, to the more severe side effects, including agitation, confusion, delirium, and seizures [68].

Deprescribing is the systematic evaluation of a patient’s medications with the goal of identifying medications that may be inappropriate for the patient for any number of reasons, such as lack of need for that medication, duplicative prescribing, or medications for which the risk to the patient outweighs the potential benefit [69]. In some cases, safer but similarly effective alternative drugs exist. For seniors, there are entire drug classes that are considered inappropriate; for most seniors, these include anticholinergics, benzodiazepines, antipsychotics, opioids, and proton pump inhibitors because of their association with potentially severe adverse events. Deprescribing must be done systematically, prudently, and under close clinical supervision with full patient or caregiver cooperation. Caution is warranted as withdrawal symptoms are possible with some drugs. In the case of opioids and benzodiazepines, for example, a tapering plan is advised, with slow tapers preferred to rapid ones [70, 71]. In some cases, it is advisable for the patient to participate in the decision-making process with the clinical team and help direct the speed of the taper rather than rely on a calendar plan [72, 73]. Deprescribing should be considered in all patients taking high-risk medications or patients nearing the end of life. Deprescribing in the elderly should be considered with dementia, frailty, and disease progression. For patients of all ages taking multiple medications and reporting new side effects, deprescribing should be carefully considered and discontinuation of certain drug(s) may be beneficial. Sound clinical judgment must be exercised, because there are cases in which a potentially dangerous medication cannot readily be discontinued or must be taken in the best interests of the patient [74]. In cases where the patient is able to participate, shared decision-making models on deprescribing and tapers can be beneficial [75]. In all instances, patients should be encouraged and supported throughout the process.

Clinical considerations

There are important clinical considerations to help mitigate or prevent iatrogenic effects of pain treatments. While complications in interventional procedures are relatively rare, all spinal procedures should be performed under fluoroscopic guidance. The decision to use injections should be preceded by magnetic resonance imaging (MRI), which will help determine the level of the injection. If arterial blood is encountered during the injection itself, it is prudent to consider aborting the procedure. While clinicians are well advised to take all appropriate and required steps, doing “everything possible” may cause over-management and its own complications.

Epidural abscesses are rare but potentially life-threatening iatrogenic side effects of epidural injections of corticosteroids. MRI is the preferred imaging mode for diagnosing such abscesses, but computed tomography plus myelography may be used if an MRI is impossible. When conducting imaging studies, it is crucial to note that these abscesses can be multifocal [76]. At greatest risk for these abscesses are immunocompromised patients, especially patients with diabetes. The most common pathogen involved is *S. aureus*. Before administering such an injection, consider the hematological and oncological clearance of the patient and do not inject if there is evidence of any pre-existing systemic infection.

Educate interventional pain patients to consult with the physician and the dentist before undergoing dental procedures. It has been speculated that individuals at elevated risk for epidural abscesses, such as those with compromised immune systems, be treated with prophylactic antibiotics prior to certain dental procedures, but there is no clear guidance in the literature on that point [77]. Epidural abscesses can be challenging to diagnose and are sometimes erroneously attributed to injection site pain or injection-related effects. There is extreme tenderness at and surrounding the injection site with an epidural abscess even when the entry site appears normal. Not all patients with an epidural abscess will present with higher than normal white blood cell counts and fever, although many do [76].

For transforaminal epidural injections of steroids into the cervical spine, computed tomography imaging studies or Doppler ultrasound evaluations should be carried out in order to prevent vessel injury. This will allow the visualization of potentially vulnerable vessels [78]. Radial nerve injuries due to upper-extremity injections may require surgical intervention, with the encouragement that early diagnosis and intervention offers an opportunity for favorable outcome [79, 80].

Pharmacologic therapy for painful conditions has proven to be safe and effective for many patients, but sound clinical judgment and periodic re-evaluation are required for optimal results. Particularly for elderly patients and those with impaired renal or hepatic systems, certain drugs may be contraindicated. In all patients who take multiple drugs, there is a potential for pharmacokinetic drug-drug interactions [81]. Furthermore, patients with complex comorbidities, multimorbid conditions, or age-related conditions may be prescribed polypharmacy in which the potential harms outweigh the benefits. For geriatric patients, the Beers Criteria, STOPP-START criteria, and the anticholinergic drug scale can be beneficial in determining which drug(s) are appropriate for this population. Side effects can occur with analgesic and other therapies, and these adverse events may in some cases be severe and even potentially life threatening.

Of course, sound clinical judgment is required, particularly for patients with mental health conditions, those at the end of life, or those with extremely complex and life-threatening diseases. Since many patients are prescribed drugs without taking a “big picture” view of their medications and a holistic assessment of their health and function, it is important to review medications on a regular basis [3]. Where medications are no longer necessary, cause treatment-limiting side effects, are contraindicated, or have the potential to interact with other more necessary drugs, a deprescribing regimen may be appropriate. Deprescribing should always be undertaken systematically. There are many drugs, such as opioids and benzodiazepines, which should not be discontinued abruptly but may require a taper plan [82]. Tapering is an incremental program designed to gradually reduce the dose of a given drug until it can be discontinued entirely. While tapering plans have been published, tapering is often highly individual as some patients may require slower tapers or more time “plateauing” at specific doses than others [83]. For example, if an opioid is to be discontinued, the patient should be tapered gradually to mitigate or eliminate withdrawal symptoms, but the clinician will also need to continue to provide a pain-control regimen [84]. This may be a new, safer form of drug therapy or the use of other techniques. When the patient can participate in clinical decision-making, it is prudent to include them in the deprescribing process and taper plans [75, 85].

## Conclusions

Despite progress in treating painful conditions, interventional procedures and drug therapy to reduce pain may introduce iatrogenic side effects. These adverse events range from those that are merely bothersome to those that reduce the quality of life or even pose threats to life. Pain physicians must be cognizant of the rare but potentially catastrophic iatrogenic events that may occur in interventional pain treatments. Likewise, drug therapy may cause serious side effects, such as opioid-induced constipation which requires judicious management, ideally with a PAMORA product. Geriatric patients and those with complex conditions may require periodical medication reviews to avoid complications associated with polypharmacy, prescribing cascades, and the use of contraindicated or suboptimal drugs. When deprescribing a patient taking polypharmacy, care should be taken to avoid or mitigate withdrawal symptoms, typically using a validated taper plan and a shared decision-making model with the patient.
